# Breaking the data cage in generative materials discovery

**DOI:** 10.1093/nsr/nwag193

**Published:** 2026-03-26

**Authors:** Shuaihua Lu, Xiao Cheng Zeng

**Affiliations:** Department of Materials Science and Engineering, City University of Hong Kong, China; Department of Materials Science and Engineering, City University of Hong Kong, China

**Figure 1.**
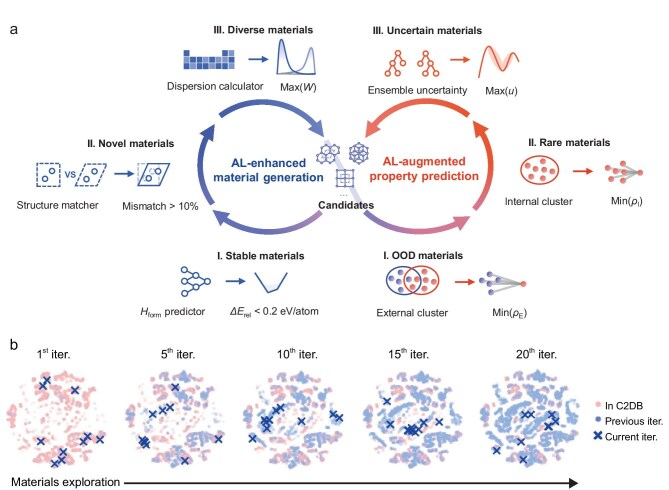
(a) Schematic of the DuALGen framework. The architecture couples two active learning loops: a generative loop for autonomous discovery and density functional theory (DFT) validation of novel 2D materials, and a predictive loop for refining property models through out-of-distribution sampling. This dual-loop iteration enables continuous exploration of the chemical space while systematically correcting for data bias. (b) Progressive evolution of the chemical space. t-distributed stochastic neighbor embedding (t-SNE) visualization illustrates the expansion of the latent space across iterations (iter.). The generated candidates (blue symbols) significantly enrich the diversity of the design space, effectively filling underpopulated regions in the original Computational 2D Materials Database (C2DB) dataset (pink dots).

Generative artificial intelligence (GAI) is rapidly reshaping materials discovery, offering access to vast and previously unexplored regions of chemical space [1,2]. By learning from existing data, generative models are expected to extrapolate beyond known materials and discover new ones that may transcend human intuition. In practice, however, this capability remains constrained by ‘historical data bias’, with models tending to revisit well-explored regions of known materials, thus limiting true novelty. This limitation is not merely algorithmic, but rooted in the nature of materials data itself, which is shaped by experimental feasibility, thermodynamic stability, and historical research priorities, resulting in uneven coverage of chemical space and the amplification of bias in generated outputs [3]. More importantly, while generated materials are increasingly explored for their functional potential, a systematic understanding of how generated materials differ from known ones, and how such differences influence both predictive modeling and the generative process itself, remains largely incomplete. To address this gap, Wang and co-workers introduce a dual active-learning (AL) framework, DuALGen (Fig. 1a) [4]. Its central idea is to couple two complementary closed loops that enable sustained and adaptive exploration of chemical space. The generative loop expands the design space through multi-objective sampling that balances stability, novelty, and diversity, while the predictive loop continuously incorporates underrepresented candidates to correct distributional bias and improve model generalization. Through this interplay, DuALGen moves beyond static, one-shot generation toward a self-evolving discovery process.

This bidirectional closed-loop framework not only enables the construction of a large-scale database of >10 000 stable 2D compounds (Gen2DB), but also reveals a range of material candidates with promising properties such as high carrier mobility, wide bandgap, and intrinsic magnetism, highlighting their application potential. Beyond these applications, it further provides a basis to analyze the divergence between generated and known materials. By understanding such divergence and adopting targeted sampling, the framework enables systematic utilization of generated data, thereby enhancing both the exploratory capacity of the generative model and extrapolative capability of the predictive model. In doing so, it drives the continuous expansion of chemical space and unlocks the discovery of novel 2D materials beyond known regimes (Fig. 1b).

In summary, this theoretical work develops an active-learning framework together with a generative model, enabling efficient exploration of chemical space and yielding a large set of stable 2D materials. It reframes generative material design as a data-centric, self-evolving, and closed-loop paradigm, in which data, models, and, potentially, experimental efforts co-evolve to enable the discovery of genuinely novel candidates with transformative potential.
